# Whole-Brain Network Connectivity Underlying the Human Speech Articulation as Emerged Integrating Direct Electric Stimulation, Resting State fMRI and Tractography

**DOI:** 10.3389/fnhum.2018.00405

**Published:** 2018-10-11

**Authors:** Domenico Zacà, Francesco Corsini, Umberto Rozzanigo, Monica Dallabona, Paolo Avesani, Luciano Annicchiarico, Luca Zigiotto, Giovanna Faraca, Franco Chioffi, Jorge Jovicich, Silvio Sarubbo

**Affiliations:** ^1^Center for Mind/Brain Sciences (CIMeC), University of Trento, Trento, Italy; ^2^Division of Neurosurgery, “S. Chiara” Hospital, Azienda Provinciale per i Servizi Sanitari, Trento, Italy; ^3^Structural and Functional Connectivity Lab (SFC-Lab) Project, Division of Neurosurgery, “S. Chiara” Hospital, Azienda Provinciale per i Servizi Sanitari, Trento, Italy; ^4^Department of Radiology, Neuroradiology Unit, “S. Chiara” Hospital, Azienda Provinciale per i Servizi Sanitari, Trento, Italy; ^5^NiLab, Bruno Kessler Foundation - FBK, Trento, Italy; ^6^Department of Neurosciences, Biomedicine and Movement Sciences, Section of Neurosurgery, University of Verona, Verona, Italy

**Keywords:** speech, articulation, network, functional connectivity, resting state fMRI, tractography

## Abstract

Production of fluent speech in humans is based on a precise and coordinated articulation of sounds. A speech articulation network (SAN) has been observed in multiple brain studies typically using either neuroimaging or direct electrical stimulation (DES), thus giving limited knowledge about the whole brain structural and functional organization of this network. In this study, seven right-handed patients underwent awake surgery resection of low-grade gliomas (4) and cavernous angiomas. We combined pre-surgical resting state fMRI (rs-fMRI) and diffusion MRI together with speech arrest sites obtained intra-operatively with DES to address the following goals: (i) determine the cortical areas contributing to the intrinsic functional SAN using the speech arrest sites as functional seeds for rs-fMRI; (ii) evaluate the relative contribution of gray matter terminations from the two major language dorsal stream bundles, the superior longitudinal fasciculus (SLF III) and the arcuate fasciculus (AF); and (iii) evaluate the possible pre-surgical prediction of SAN with rs-fMRI. In all these right-handed patients the intrinsic functional SAN included frontal, inferior parietal, temporal, and insular regions symmetrically and bilaterally distributed across the two hemispheres regardless of the side (four right) of speech arrest evocation. The SLF III provided a much higher density of terminations in the cortical regions of SAN in respect to AF. Pre-surgical rs-fMRI data demonstrated moderate ability to predict the SAN. The set of functional and structural data provided in this multimodal study characterized, at a whole-brain level, a distributed and bi-hemispherical network subserving speech articulation.

## Introduction

Speech articulation is the process of producing individual sounds to compose a word (Levelt, 1993). According to the articulatory loop model by Baddeley (1990), speech articulation is composed by a planning phase in which sounds sequences are retrieved followed by an execution phase which involves the corresponding motor plans to produce desired words. The speech articulatory loop thus constitutes the final and essential stage for encoding articulatory programs into language production. Understanding the network of brain areas involved in the speech articulatory loop is crucial for basic modeling of speech production (Hickok and Poeppel, 2004; Saur et al., 2008; Fridriksson et al., 2016) and its disorders (Duffy, 2016; Boschi et al., 2017; Brabenec et al., 2017; Cordella et al., 2017), as well as for reliable identification of key speech articulation areas to be avoided during surgical resections (Mandonnet and Duffau, 2017).

Speech arrest is defined as discontinuation in number counting but with voluntary movements of tongue being possible (anarthria) (Sanai et al., 2008; Mandonnet et al., 2017). Because a counting task minimally requires to process semantic, syntactic, or phonological information, the detection of this deficit provides a very specific tool to disrupt speech articulation (Price, 2010). The counting test is, in fact, commonly adopted worldwide to map and preserve the speech articulation functional network during surgery for resection of intra-cerebral lesions in critical regions independently from the language lateralization (Pallud et al., 2018). Considering the high-reproducibility of speech arrest localization by direct electrical stimulation (DES) in the ventral pre-motor cortex (VPMC), it is routinely used to assess the amplitude threshold for intra-operative mapping during awake surgery (Freyschlag and Duffau, 2014; van Geemen et al., 2014; Mandonnet et al., 2017).

It has been demonstrated that combining brain stimulation with neuroimaging techniques in a “perturb and measure” approach allows mapping distributed brain activity related to the perturbation of a cortical region (Paus, 2005). Therefore measuring patterns of activation related to speech arrest may provide a tool to map the network of functionally and structurally connected cortical regions (hubs) supporting speech articulation (Sporns, 2013).

With respect to functional connectivity of speech articulation, a recent invasive study characterized the temporal evolution of this network using intracranial recording and stimulation on 10 right handed pediatric epilepsy patients responding a tone listening and repetition task (Nishida et al., 2017). The authors recorded cortical–cortical evoked potential in the inferior frontal gyrus (IFG) and superior temporal gyrus (STG) following stimulation of the precentral gyrus (PCG) that caused speech arrest in the investigated hemisphere (five left, five right). In a different non-invasive study, areas subserving speech arrest were investigated with repetitive transcranial magnetic stimulation (rTMS) in combination with language task fMRI (Kononen et al., 2015). The authors found a bilateral role of the PCG for speech arrest with rTMS regardless of subjects’ handedness (Kononen et al., 2015). However, the covert (internal speech) language fMRI task yielded brain activation overlapping with the areas of speech arrest only in the dominant hemisphere (Kononen et al., 2015). The limited brain coverage of intracranial recording (a few regions within one hemisphere) together with the choice of a strong language lateralizing fMRI task (internal speech) combined with the TMS measures may have not allowed to detect all the cortical areas recruited for speech articulation and, in particular, to detect whether mirror areas across the two hemispheres are involved. This last hypothesis (i.e., bi-hemispherical organization of speech articulation functional connectivity) is indeed supported by the findings of intra-operative mapping studies that have consistently detected speech arrest on both right and left VPMC in right handed patients (Mayka et al., 2006; Chang et al., 2011; Tate et al., 2014).

With regard to structural connectivity of speech articulation a series of studies have reported that intra-operative subcortical stimulation of both the anterior portion of the superior longitudinal fasciculus (SLF III) and the arcuate fasciculus (AF)cause speech arrest (Duffau et al., 2003; van Geemen et al., 2014; Sarubbo et al., 2016). However, it is not clear which among these two tracts plays the most critical functional role for speech articulation. In a study aiming to provide a subcortical atlas of human brain functions (Sarubbo et al., 2015) the authors compared the distance between the centers of mass of several functional response errors collected at DES and the white matter (WM) bundles included in a DTI Atlas (Thiebaut de Schotten et al., 2011). They found that the center of mass of subcortical speech arrest points collected in 22 patients was closer to the SLF III than the AF. This result suggests that SLF III may provide the main tract subserving speech articulation. However, to prove this hypothesis it remains to be demonstrated that this bundle is the main pathway of structural connectivity between the cortical areas recruited in speech articulation at a single patient level.

Based on the findings of above mentioned studies we hypothesized that the structural and functional speech articulation network (SAN) could be characterized at the level of the whole brain in single-subject data by combining pre-surgical resting state fMRI (rs-fMRI) and diffusion-weighted MRI tractography data together with information about speech arrest sites obtained with DES during routine awake surgery. Therefore, we collected and performed a combined analysis of these data in a group of patients with brain tumor and cavernous angiomas with the aim of: mapping the SAN at a whole brain level calculating the rs-fMRI functional connectivity of the speech arrest sites detected with DES with all the other regions of the brain; determining which among the SLF III and AF provides the major associative tract for speech articulation by comparing their density of cortical terminations in the regions of the SAN; evaluating the extent to which pre-operative rs-fMRI networks obtained with independent component analysis (ICA) can predict the SAN as determined using the intra-operative speech arrest seed. This information, in particular, may be helpful if rs-fMRI would be used for pre-surgical mapping of speech articulation areas.

## Materials and Methods

### Participants

MRI and intra-operative DES data were acquired and retrospectively analyzed on seven right-handed patients (four males; age range 36–48 years, mean ± SD = 41.4 ± 5.4 years) submitted to routinely surgical removal of low grade gliomas (4) and cavernous angiomas with DES cortico-subcortical mapping. **Table 1** reports age, gender, lesion location and histology (in case of gliomas) for each patient included in the study. All the surgical (i.e., awake surgery with DES) and neuroradiological (i.e., diffusion and rs-fMRI imaging) procedures performed and described in this study are part of the standard protocol in our Center for pre-operative planning and resections of lesions harboring eloquent areas. All the participants provided an informed consent to the collection of the data and the use for scientific purposes.

**Table 1:** Clinical and demographic data of patients included in this study. All subjects were right-handed.

Case	Age	Tumor location	Histology
1	37	R Frontal	Grade II Astrocytoma
2	45	R Supplementary Motor Area	Grade II oligodendroglioma
3	39	L Frontal	Cavernoma
4	48	R Frontal	Grade II Astrocytoma
5	37	L Frontal	Cavernoma
6	36	L Temporal	Grade II Astrocytoma
7	48	R Frontal	Cavernoma

*L/R, left/right brain hemispheres.*

### MRI Data

All the patients underwent to a routinely protocol at the “S. Chiara” Hospital of Trento APSS for pre-operative planning for resection of lesions in critical areas, including diffusion-weighted imaging (DWI) for tractography and rs-fMRI. Images were acquired using a clinical Optima MR450w GE 1.5 T scanner (GE Healthcare, Milwaukee, WI, United States) equipped with an 8-channel receive head RF coil.

The pre-surgical imaging protocol included a 3D T1-weighted inversion recovery gradient echo sequence for structural imaging (axial acquisition, TR/TI/TE = 10.64/450/4.23 ms, FA = 12°, square field of view (FOV) = 256 mm, voxel size = 1 × 1 × 1 mm^3^, ASSET acceleration factor = 2) and a 2D T2^∗^-weighted gradient echo planar imaging sequence for rs-fMRI (axial acquisition with ascending interleaved slice order, TR/TE = 2600/45 ms, FA = 87°, square FOV = 256 mm, voxel size = 4 × 4 × 4 mm^3^, slice gap = 0.8 mm, fat saturation, ASSET acceleration factor = 2, 275 volumes). During the acquisition of rs-fMRI data patients were asked to lay still with eyes open.

For tractography, a 60-direction DWI scheme was acquired (one acquisition) using a single-shot multislice spin echo–echo planar sequence with the following attributes: 50 slices; square FOV: 240 mm; voxel size = 2.4 × 2.4 × 2.4 mm^3^; TR/TE = 13000/95.8 ms; flip angle: 90°; b values of 0 and 1,000 s/mm^2^.

For additional tumor characterization, at the end of the MR protocol a second 3D T1-weighted image was acquired (identical to the previously described) following injection of gadolinium. During the procedure of DES, the spatial coordinates of the speech arrest sites are saved using this post gadolinium structural image as reference.

### Resting State fMRI Preprocessing

Resting state fMRI data preprocessing was performed using SPM12, FSL (version 5.09), and custom based MATLAB code (version 8.1.0) following Minati et al. (2014). The preprocessing steps are outlined in **Supplementary Figure S1** and consisted of: removal of the first four EPI volumes to allow the signal to reach steady state magnetization, slice timing and head motion correction, median (*r* = 2) filtering, de-trending with a fourth order polynomial and low pass filtering with second-order Butterworth filter (*f* < 0.1 Hz). Head motion shift and rotation parameters and the average time series of WM and cerebrospinal fluid (CSF) tissue masks, obtained from the segmentation and co-registration of the pre Gad T1-weighted images to the rs-fMRI data, were temporally filtered as above and regressed out. Volume outliers were inspected (after head motion correction) and removed using the ArtRepair software version Version 5b^1^. A volume was classified as an outlier either if its frame by frame head motion was greater than 0.5 mm or its whole brain average time series value was above 2.5 standard deviation the average global signal intensity and it was replaced by interpolating the values from the preceding and subsequent volumes. Finally before iFC analysis rs-fMRI data was spatially smoothed using an 8 mm Full Width Half Maximum Gaussian filter.

### Diffusion Processing and Tractography

The processing of DWI was performed concatenating a step for pre-processing, a step for voxel model reconstruction, and a step for deterministic tractography. The analyses pipeline was implemented using FSL and Dipy, an open source library for the analysis of diffusion MRI data (Jenkinson et al., 2002; Garyfallidis et al., 2014).

Following eddy current and head motion correction, images were co-registered to the structural post-Gad 3D T1 and the diffusion tensor model was applied to reconstruct the main orientation of diffusivity for each voxel. The subsequent step of tracking was based on the Euler Delta Crossing (EuDX) method, with 106 seeds. The resulting whole-brain tractograms consist of approximately 150 thousand streamlines. The bundles included in this study (i.e., SLF III and AF) were tracked with TrackVis^2^ and a multiple (inclusion/exclusion) regions of interest (ROIs) approach by an expert brain anatomist (author, SS), as part of the pre-surgical planning and for intra-operative support in neuronavigation. For AF an inclusion ROI was placed at the stem (in the anterior and lateral para-trigonal space), posteriorly to the posterior sulcus of the insula and below the posterior thirds of STG and middle temporal gyrus (MTG) and an exclusion ROI was placed at the level of the stem of SLF III, in the lateral WM at the most ventral border between frontal and parietal lobe (Sarubbo et al., 2016). Vice versa, the AF stem ROI was used with “no part” logical operator for tracking the SLF III. U-fibers (defined as fibers connecting adjacent gyri) and the posterior indirect component of the SLF were removed.

### Surgical Procedure: Intra-operative Functional Mapping and Monitoring

All patients underwent asleep–awake–asleep surgery with total intra-venous anesthesia using Remifentanil and Propofol infusion stopped before the awake mapping and neuropsychological monitoring. Cortico-subcortical DES was performed to obtain the most reliable mapping of eloquent structures (Coello et al., 2013). A bipolar electrode with 7 mm spaced tips delivering a biphasic current (pulse frequency of 60 Hz; single-pulse phase duration of 1 ms; amplitude range: 2–4 mA) was used. During the mapping, the intensity threshold was set by evoking a speech arrest (with alternated and regular tongue movements being possible) while the patient was performing a counting task (from 0 to 10), accordingly to previous reports and protocols (Tate et al., 2014; Sarubbo et al., 2015; Riva et al., 2016; Mandonnet et al., 2017). Following speech arrest in all the cases motor mapping was performed for searching for a motor response (facial or tongue) to identify the primary motor cortex and then a full cortical mapping was performed with denomination object and Pyramids and Palm Tree Test (Bello et al., 2006; Coello et al., 2013). In the four patients operated on the right hemisphere after localizing speech arrest sites any other aspect of language responses (i.e., anomia, semantic and phonological paraphasia) was tested. After the cortical mapping the brain tissue resection started, with patients performing continuously the tasks, and it was stopped when the expected functional limits were reached. The different functional response errors collected during intra-operative mapping were associated with a specific tag, stored with high-resolution pictures and subsequently included on the post-gadolinium preoperative T1-weighted images by comparing the anatomical landmarks of the single pictures with the axial volumetric T1-weighted and the reformatted sagittal and coronal images oriented according to the intra-operative lateral position of the head (Tate et al., 2014; Sarubbo et al., 2015). Each functional response was saved as a separate ROI.

### Speech Arrest Seed-Based Functional Connectivity Maps

The goal of this analysis was to generate a group intrinsic (i.e., at rest) functional connectivity (iFC) map of the SAN using for each subject the speech arrest sites as ROI seeds for the functional connectivity. For each patient a seed based iFC map of the SAN was generated according to the following pipeline: a 7 mm radius sphere was drawn from the geometrical center of the speech arrest ROI aligned with the T1-weighted post-gadolinium images. The six degree of freedom rigid body matrix to register the T1-weighted post-gadolinium to the T1-weighted (pre-gadolinium) images was determined using FSL-FLIRT. This matrix was subsequently composed with the one calculated to co-register the pre-gadolinium T1-weighted images to the rs-fMRI data and applied it to the 7 mm radius speech arrest sphere. The Pearson’s correlation coefficient was calculated between each voxel of the pre-processed rs-fMRI dataset and the speech arrest ROI average time series and converted to z-score using the Fisher transform. For each patient the obtained cross correlation map was thresholded at *z* = 0.21, *p* = 0.01 single voxel and family wise error corrected at a significant level of *p* < 0.05 using cluster-based thresholding based on cluster-size parameters estimated by AFNI’s 3dClustsim (Forman et al., 1995).

In order to assess the consistency and variability of the speech articulation iFC across patients, a voxel-wise map of iFC agreement frequency across subjects was created on the Montreal Neurological Institute (MNI) space. To this purpose each subject’s thresholded speech articulation iFC map (*z* > 0.21) was warped into MNI space (FSL T1 template, 2 × 2 × 2 mm^3^). The co-registration matrix was determined by multiplying the inverse of the matrix obtained to co-register the pre-gadolinium T1-weighted images to the motion corrected rs-fMRI data with a 12 degree of freedom matrix calculated using FSL-FLIRT to register the pre-gadolinium T1-weighted images to the FSL T1 template, 2 × 2 × 2 mm^3^. The quality of the co-registration was visually assessed and deemed adequate for each patient by the first author. Finally, as a measure of SAN consistency across subjects, the proportion of patients for which each voxel of the FSL template brain was included in the speech articulation iFC map was calculated (Seghier and Price, 2016). The average group network consistency within the gray matter (GM) ROIs of the anatomical automatic labeling atlas (AAL) atlas was also derived (Tzourio-Mazoyer et al., 2002).

### Relationships Between Functional Regions of SAN and Tractography Reconstructions

The goal of this analysis was to determine, for each subject, the topographic relation between the cortical terminations of the SLF III and AF WM bundles and the subject’s specific functional SAN. The underlying question was to test whether one of these two key language tracts was more strongly associated with the articulatory loop than the other. For each patient a 12 parameters affine transformation was determined using FSL-FLIRT to register the T1 post-gadolinium weighted images to the MNI space and applied to the reconstructed SLF III and AF tracts. Following Hau et al. (2016) for each region of the AAL atlas included in the group SAN (iFC cluster sites, see Results) a termination percentage score (TPS) was calculated, dividing the total number of tract streamlines ending in it by the total number of streamlines ending in the GM regions of the AAL atlas (multiplied by 100) for both SLF III and AF. Subsequently, because each patient had only the lesion hemisphere SLF III and AF pre-operatively reconstructed, the left and right portion of each iFC cluster sites were combined and the median TPS across the patients in each of the iFC cluster sites where the frequency of tract terminations was >0 (i.e., at least one subject) was calculated. A Wilcoxon test was run to assess whether the distribution of cortical terminations for the two bundles in the iFC cluster sites was statistically significantly different.

### Pre-operative Determination of the Functional SAN

It would be useful if pre-surgical rs-fMRI data could be used to help the neurosurgeon anticipate prior to surgery, the subject’s specific functional network related to speech arrest sites. The goal of this analysis was therefore to compare the spatial correspondence between the functional SAN derived from the speech arrest seeds with the ICA networks that were predicted prior to the intervention.

For each patient the preprocessed rs-fMRI dataset was decomposed into 20, 30, and a number of components (range 29–39) determined by probabilistic ICA implemented in MELODIC-FSL (Beckmann et al., 2005). For each component a voxel-wise z-score map was obtained reflecting the agreement between the time series of each voxel with the time series of the specific component. Each z-score map was warped in MNI space using the previously determined registration matrix to warp the seed based iFC maps. Subsequently, following Van Dijk et al. (2009), an automated template matching procedure was applied to select the component that best fit a binary template of the SAN. This template was defined selecting the voxels of FSL template brain that were included in the SAN group frequency iFC map (see Results). Specifically, for each component the difference between the average z-score of all the voxels inside the template and the average z-score of all voxels outside the template was calculated and the one in which this difference (the goodness of fit-GOF) was the greatest was selected. The Spearman’s correlation coefficient between the GOF of the component best matching the speech articulation template and the average correlation strength of the speech arrest seed based iFC map inside the template was calculated. Finally, a Kruskal–Wallis’s test, was run to assess whether the degree of similarity (highest GOF) between the ICA and seed-based speech articulation iFC maps was affected by the number of ICs used (20, 30, pICA determined).

## Results

### Intrinsic Functional Connectivity Mapping of the SAN

**FIGURE 1:**
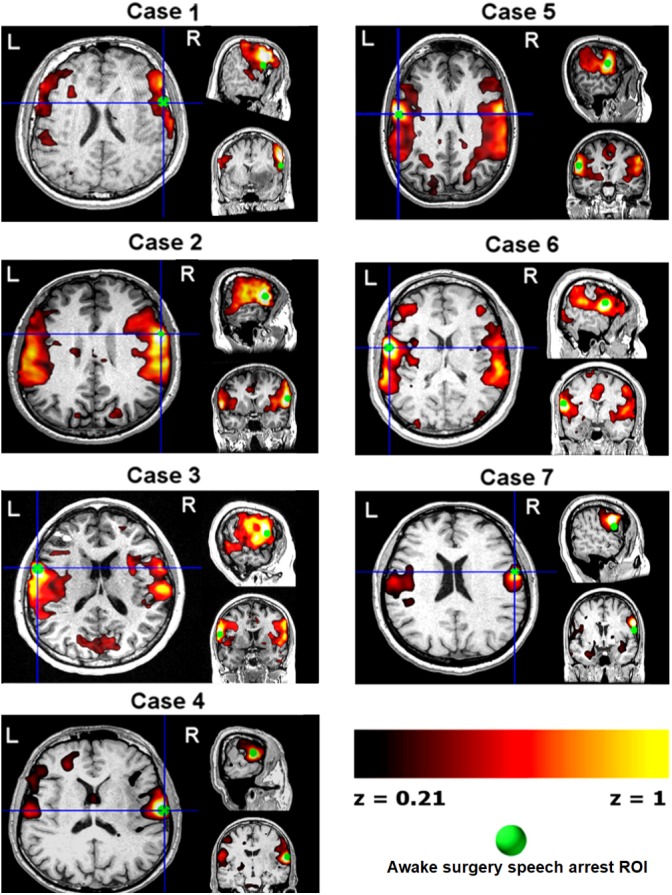
Single-subject pre-surgical resting state functional MRI connectivity networks (*z* > 0.21, hot color scale) using as seed each patient’s speech arrest location (green ROIs, **Table 2** and **Figure 3**) obtained during awake surgery. Data are shown in each subject’s space. L/R, left/right brain hemispheres.

**FIGURE 2:**
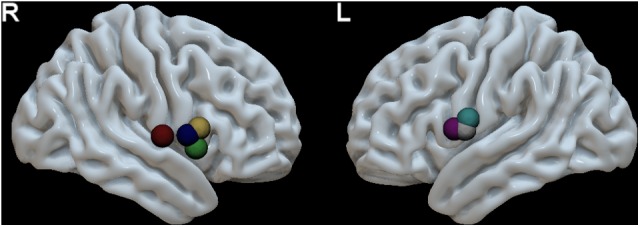
MNI space localization of each patient’s speech arrest ROI obtained during awake surgery using direct electric stimulation (see **Table 2** for MNI coordinates). These ROIs were used as seeds in pre-surgical resting state fMRI data to calculate the speech articulation functional connectivity map (**Figure 1**). The speech arrest ROIs are denoted with a different color for each different patient. R, right hemisphere (four patients) and L, left hemisphere (three patients).

The speech arrest seed-based iFC maps for each patient are shown in **Figure 1**. The speech arrest sites were mainly located in right and left frontal inferior and rolandic opercula, in VPMC, according to previous literature findings on the intra-operative detection of anarthria (i.e., interruption of speech articulation not due to tongue movement impairment) (Chang et al., 2011; Tate et al., 2014). We report the coordinates (**Table 2**) of each speech arrest ROI center in MNI space (**Figure 2**). The SAN was consistently and bilaterally observed across all patients in the fronto-ventral, temporal, sensorimotor and inferior parietal lobule regions, regardless of whether speech arrest sites were localized in the left (three cases) or right (four cases) brain hemispheres.

**Table 2:** Speech arrest ROIs center coordinates in MNI space (mm, origin in the anterior commissure, LPI orientation).

Case	Brain region	X	Y	Z
1	R Frontal Inferior Operculum	58	14	16
2	R Frontal Inferior Operculum	62	12	4
3	L Frontal Inferior Operculum	−62	8	12
4	R Rolandic Operculum	66	−8	12
5	L Rolandic Operculum	−62	2	12
6	L Postcentral	−62	0	18
7	R Rolandic Operculum	62	8	12

*See also **Figure 2** for a visualization of the seeds in MNI space. L/R, left/right brain hemispheres.*

**FIGURE 3:**
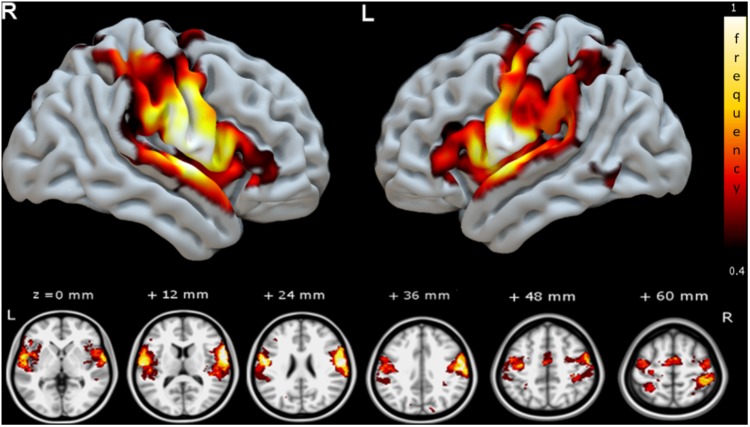
Group speech articulation functional connectivity network shown as voxel-wise group consistency map of spatial overlap in MNI space (color bar represents % of subjects with common SAN voxel). The frequency map of spatial overlap (thresholded at >40%, or at least three subjects from the group of seven) is shown on the MNI cortical surface (upper row) and axial slices (lower row). Regions with highest speech planning network consistency across patients are distributed bilaterally in fronto-temporal and parietal areas. R, right hemisphere and L, left hemisphere.

In **Supplementary Figure S2** the group functional connectivity SAN are shown as voxel-wise frequency maps in MNI space at four different frequency threshold (10, 20, 30, and 40%). We determined that the group maps thresholded at 40% (i.e., at least three or more patients from the group of seven, **Figure 3**) could reliably represent a group functional connectivity SAN because it includes on both hemispheres cortical regions known to be involved in speech articulation (Basilakos et al., 2017) while the additional areas appearing at lower thresholds may be attributed to the inter-subject variability from the various noise sources in rs-fMRI signal (Birn, 2012; Murphy et al., 2013; Power et al., 2015). The GM AAL atlas regions included in this map are listed in **Table 3**. Group consistency of the functional SAN was observed bilaterally in speech-specific auditory processing (middle and posterior STG), working memory/language articulation (inferior frontal cortex, supramarginal gyrus [SMG]) and motor articulation (sensorimotor cortex) areas.

**Table 3:** AAL atlas regions with the highest average group frequency (>40%) overlap with the functional speech articulatory network as defined by the seed-based analysis on speech arrest ROIs (**Figure 3**).

Regions	Average frequency value
Rolandic Operculum L	0.70
Rolandic Operculum R	0.67
Heschl’s L	0.61
Frontal Inferior Operculum R	0.55
Frontal Inferior Operculum L	0.55
Temporal Superior L	0.54
SupraMarginal L	0.54
Postcentral R	0.52
SupraMarginal R	0.49
Heschl’s R	0.48
Postcentral L	0.45
Temporal Superior R	0.45
Precentral L	0.44
Insula L	0.44
Insula R	0.44
Precentral R	0.42

*L/R, left/right brain hemispheres.*

### Spatial Correspondence Between the Functional Connectivity SAN and GM Terminations of SLF III and AF White Matter

**FIGURE 4:**
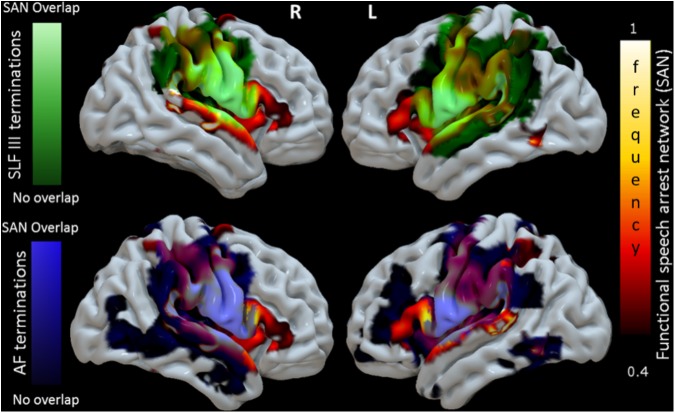
3D reconstruction of correspondence between the functional connectivity speech articulatory network, SAN (shown in **Figure 3**, here hot scale) and cortical terminations of two major WM language tracts in MNI space. The upper panel shows the cortical terminations of the SLF III (green). Terminations not overlapping the SAN appear in dark green, while those overlapping are shown in light green. The lower panel shows the corresponding results for the terminations of the AF, in blue. For visualization purposes the degree of overlap was calculated voxel/vertex wise first binarizing the density cortical terminations maps and then adding the speech arrest network frequency value in the same voxel. The results of the quantitative analysis of the cortical termination density maps in the regions of the SAN are presented in **Figure 5**.

**FIGURE 5:**
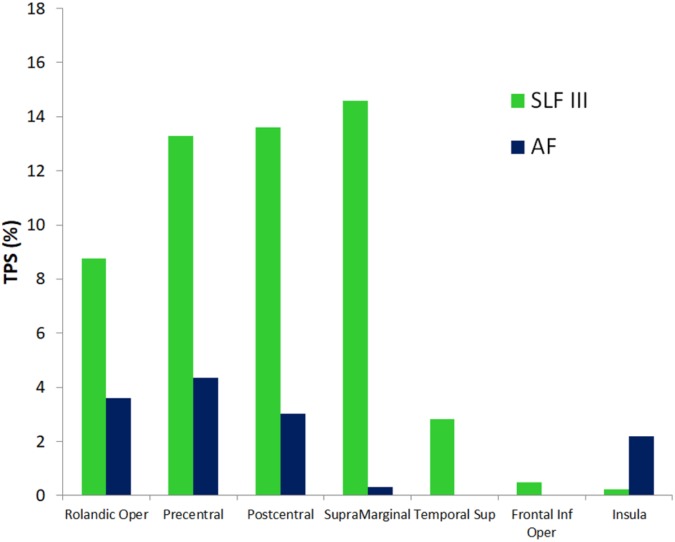
Quantitative correspondence between the cortical terminations density (termination percentage score, TPS) of SLF III and AF into the gray matter regions of the SAN cluster sites with highest consistency across patients (>40%, **Figure 3** and **Table 3**). Only cluster sites with density > 0 are represented. See section “Spatial correspondence between the functional connectivity SAN and GM terminations of SLF III and AF white matter” for more details.

**Figure 4** shows a 3D surface representation of the overlap between the SLF III and AF cortical terminations maps (median across subjects) and the group iFC of speech articulation thresholded at 0.4, as in **Figure 3**. The analysis of the distribution of cortical terminations across the iFC sites overall showed (**Figure 5**) a much higher density of the SLF III (range 0.22–14.60%) in comparison with the AF (0.30–4.35%), according also to the results of the statistical analysis (Wilcoxon test *z* = 2.02, *p* = 0.04, and *n* = 7). Specifically the SLF III terminated predominantly in the most ventral and anterior parietal cortices (14.6% for SMG and 13.6% for PostCG) followed by the most ventroposterior cortices of the frontal lobe (13.29% for PCG and 8.75% for rolandic opercula). Minor branches projected toward the posterior third of the STG (2.83%), frontal inferior operculum and insula (0.47 and 0.22%). The terminations of the AF in the same regions were instead observed predominantly in PCG (4.35%), Rolandic Opercula (3.59%), and the Insula (2.2%) followed by the PostCG (3.03%) and SMG (0.3%). We excluded from the analysis of the Heschl’s gyrus because median PTS was 0 for both SLF (three subject only with cortical termination range 0.07–4%) and AF (one subject PTS = 0.1% with cortical terminations).

### Pre-operative Resting State fMRI Determination of the SAN

**FIGURE 6:**
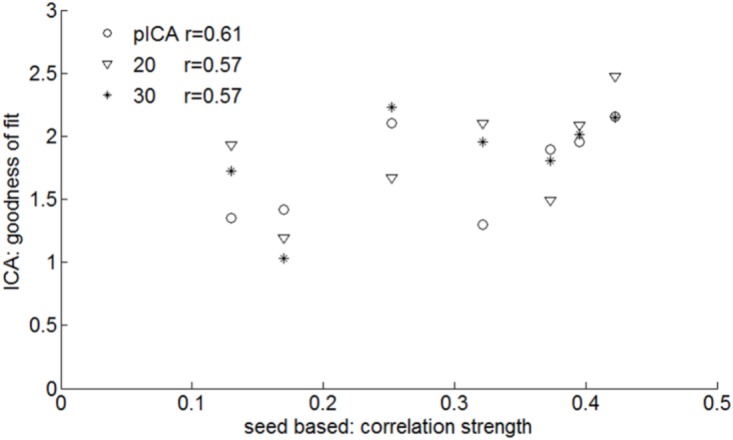
Pre-surgical resting state fMRI: similarities between ICA and speech arrest seed-based measures of the group SAN template (**Figure 3**). For each subject the ICA with the highest goodness of fit to the seed-based network template is defined as well as the average z-score within the template. The correlation between these is shown for various ICA analyses: probabilistic ICA (pICA) and deterministic ICA with 20 or 30 independent components. The highest correlation was found when the number of IC was determined by probabilistic ICA (*r* = 0.61).

Prediction of the SAN by pre-surgical rs-fMRI ICA gave promising results as evidenced by the moderate (although not significant due to the small size of our sample *N* = 7) degree of correlation between the connectivity of the SAN seed regions and the GOF of the speech articulation component obtained with ICA (**Figure 6**): *r* = 0.57, *p* = 0.2; *r* = 0.57, *p* = 0.2; and *r* = 0.61, *p* = 0.16, respectively, for 20, 30, and pICA determined number of components. **Supplementary Figure S3** shows for each patient the z-score map of the component best matching the SAN template obtained including all the voxels in the speech articulation frequency iFC map with a value higher than 40% (at least three or more patients from the group of seven). The similarity between the automatically selected (highest GOF) ICA and seed based speech articulation iFC maps was not dependent on the number of components the rs-fMRI signal was decomposed into (Kruskal–Wallis’s test *H* = 0.32, *p* = 0.85, dof = 2).

## Discussion

This study combines, for the first time to our knowledge, pre-surgical rs-fMRI and tractography data with intra-operatively defined speech arrest locations to characterize the functional and structural connectivity topography of the SAN. The main findings of the study are: (i) the intrinsic functional connectivity of the SAN includes a set of speech-specific auditory processing (middle and posterior STG), working memory/language articulation (inferior frontal cortex, SMG) and motor articulation (sensorimotor cortex) areas mirrored across the left and right hemisphere; (ii) these cortical regions are structurally connected with higher density by WM SLF III fibers; and (iii) pre-surgical rs-fMRI data provides moderate prediction of the articulatory loop network without knowing *a priori* the localization of cortical areas that, if stimulated, would impair speech articulation.

### Intrinsic Functional Connectivity of the Speech Articulation Network

One of the goals of this study was to characterize at the whole brain level the spatial pattern of the functional SAN, in particular its inter-hemispheric connectivity. To accomplish this, we used a seed-based functional connectivity analysis on pre-surgical rs-fMRI data using as seeds the speech arrest sites determined intra-operatively during standard awake surgery. The cortical areas showing group intrinsic functional connectivity included the PCG, PostCG, the rolandic opercula, pars opercularis of the IFG, SMG, insula, STG (particularly the posterior third), and Heschl’s gyrus both left and right hemisphere. Among the set of cortical areas with the highest frequency within the group (>40%), two regions, the SMG in the parietal lobe and the Heschl’s gyrus in the temporal lobe had not been previously shown to elicit neuronal response for articulation tasks in fMRI studies (Basilakos et al., 2015, 2017). The recruitment of these two areas, however, had been hypothesized by previous subcortical mapping studies that assigned a role to SLF III for speech articulation because they demonstrated how DES of this bundle induces speech arrest (Duffau et al., 2003; van Geemen et al., 2014; Sarubbo et al., 2015). The Heschl’s gyrus and SMG are indeed among the cortical regions connected by the SLF III (Duffau, 2015). Our findings therefore confirm in a multimodal perspective the actual involvement of these two cortical areas in speech articulation.

With regards to the inter-hemispheric intrinsic functional connectivity of the articulatory loop we found a consistent symmetrical bilateral segregation of the network, across right-handers explored, regardless the brain hemisphere of the speech arrest seeds. This evidence suggests, firstly, that the articulatory network is integrated only partially with the classical left-hemisphere dominated language network, as classically emerging from rs-fMRI studies (Liu et al., 2009). The absence or minimum requirement of semantic, syntactic and phonological processing for pure articulation may explain why its activation pattern is not necessarily biased on one hemisphere (Price, 2010). Secondly, our findings imply a role of the right hemisphere in speech articulation. Previous task-based fMRI and intracranial cortico-cortical studies reported activation of regions of the right hemisphere for speech articulation in right handed subjects (Basilakos et al., 2015; Nishida et al., 2017), particularly of the STG and premotor and motor cortices. However, the multimodal approach used in this work (i.e., DES sites based rs-fMRI) allows extending the results from previous studies demonstrating, consistently across subjects, iFC of the articulatory loop between homologous regions across brain hemispheres. These results are also important because they suggest, in agreement with electrophysiology studies of speech production (Brown et al., 2012, 2014; Cogan et al., 2014), that the right hemisphere should be in fact considered part of the articulatory network, even in right-handers. This finding is in contrast with the dual-stream model of auditory language processing predicting that the auditory information is transferred to the left IFG and left PCG through the dorsal pathway (Hickok and Poeppel, 2007). However, the reason why the right hemisphere had not been considered before to be involved in this model could be attributed to the covert language tasks used in the fMRI studies the dorsal stream model is based on. Indeed, in right-handed individuals these tasks have been demonstrated to provide strongly lateralized patterns of activation (Zaca et al., 2012).

### Structural Connectivity in the Speech Articulatory Network

We found that the cortical regions more frequently mapped in the speech articulatory network are structurally connected predominantly by the SLF III relative to the AF in this patient’s series.

On one hand, these findings thus provide an *in vivo* and multimodal evidence that the SLF III fibers support a functional connectivity pattern for integration of sensorial input and speech output between the temporo-parietal junction (SMG) and the ventroposterior frontal cortices (PCG and Operculi) in both left and right brain hemispheres, as previously reported with DES studies. A role in speech articulation for the SLF III has been hypothesized, in fact, starting from the seminal work by Duffau et al. (2003) demonstrating in a case report that DES of this bundle induced speech arrest. The functional role of the SLF III has been investigated more in detail by recent studies (Sarubbo et al., 2016; Papagno et al., 2017). Papagno et al. (2017) investigated the neural correlates of verbal short term memory by mapping item and order error in a digit span task and found that stimulation of the SMG and SLF III in the left hemisphere induced more order than item errors whereas stimulation of Broca’s areas had the opposite effect. They concluded that the SLF III transfers in the left hemisphere phonological store information from the SMG to speech output areas whereas the AF (not stimulated in this study) would transfer item information from Wernicke’s to Broca’s area. In a different study post-mortem dissection of SLF III was performed and the authors found that its terminations were located predominantly in the PCG, PostCG, Operculi of the IFG, and SMG (Sarubbo et al., 2016). Therefore, in relationship with these previous findings our multimodal study further supports a critical role of the SLF III for speech articulation in both brain hemispheres. In addition, these results can be considered reliable because of the correspondence between the cortical terminations of the SLF III determined from the *in vivo* diffusion MRI data and from the *ex vivo* tissue analysis (Sarubbo et al., 2016).

On the other hand, our analysis of the overall matching between these bundles’ terminations and cortical territories involved in the network is biased by the fact that AF terminate in the temporal and not in the parietal lobe. In this light our results cannot exclude a role of the AF, or at least of the more ventral fibers (Fernández-Miranda et al., 2015; Sarubbo et al., 2016) connecting the ventral and posterior cortices of the frontal lobe with the dorsal and posterior STG, as possible integration of the auditory and phonological information in the SAN.

### Resting State fMRI Prediction of the Speech Articulatory Loop

It would be potentially useful, for example in pre-surgical planning studies, to predict the SAN from the intrinsic rs-fMRI networks without prior knowledge of speech arrest sites. To this aim we assessed the degree of spatial correspondence between the pre-surgical resting state networks obtained using ICA (using different choices for the number of IC) and the intrinsic functional connectivity maps seeded in speech arrest sites found intra-operatively. We found moderate correlation (*r* = 0.57–0.61) between the two methods for calculating functional connectivity networks. Compared to the findings reported by Van Dijk et al. (2009) performing identical analysis in a study using a 3T scanner, we obtained smaller values for both the GOF of the speech articulation component obtained with ICA and the connectivity of the SAN seed regions. The explanation for this difference may be due to the lower signal to noise ratio and spatial specificity of the BOLD signal of our data acquired using a 1.5 T static magnetic field (Donahue et al., 2011).

The limited agreement between functional connectivity networks derived from ICA and seed-based results has been seen in several other studies (Joel et al., 2011; Rosazza et al., 2012). The way seed-based correlation and ICA determine functionally connected regions across the brain are conceptually different. With seed-based intrinsic connectivity analysis, networks are constructed detecting all the voxels in the brain whose signal is highly temporally correlated with the seed region average time series. In ICA, data-reduction algorithms are used to generate a set of time-courses and spatially independent maps, subsequently classified as noise or functional networks with various manual or automatic techniques (Marchitelli et al., 2016). Furthermore the differences of the results between the two techniques could be also due to a different sensitivity to the physiologically determined systematic fluctuations (Beckmann et al., 2005). Nevertheless, the comparison of these two approaches to obtain intrinsic functional connectivity maps can provide independent confirmation of findings.

To summarize, our results suggest that the SAN could be potentially obtained from pre-surgical rs-fMRI using ICA, although confirmation with a larger sample size is necessary. Furthermore, considering that speech arrest is routinely adopted as the first negative control task to define the current amplitude threshold for the whole cortico-subcortical mapping (Freyschlag and Duffau, 2014), providing SAN maps pre-operatively has the potential to guide and reduce time for intra-operative mapping and consequently to decrease the risks of co-related morbidity (Petrella et al., 2006).

### Limitations

The main limitation of this study is the small sample size (*N* = 7). However, the population of patients we investigated in this work can be considered representative of a larger sample for the following reasons: (a) the balanced distribution of the intra-operatively recorded speech errors across both hemispheres (four right and three left) in right handed patients suggests that the represented bilateral patterns of speech articulation iFC should not be due to a bias in the location of speech arrest points and (b) the inclusion of a mixed sample (i.e., LGGs and CAs), including lesions not harboring cortical and subcortical structures supposed to support this network in order to guarantee that the detected areas functionally connected are not the result of recruitment of new cortical hubs due to plasticity phenomena. In addition, none of the mapped speech arrest points was located in spatial proximity with a lesion. Therefore we do not expect that any of the iFCmap may be affected by false negative due to neurovascular uncoupling or lesion related susceptibility artifacts (Zaca et al., 2014; Agarwal et al., 2016).

In this study, the structural and functional MRI protocols, both acquisition and analysis, were kept constant for all subjects, despite the fact that we study both patients with low grade gliomas and patients with cavernous angiomas. These pathologies have different bleeding patterns and the resulting magnetic susceptibility effects may give different sensitivity to local signal T2^∗^ loss and geometric distortions.

With reference to the analysis of the WM tracts supporting speech articulation we limited our analysis to the cortical terminations of SLF III and AF. Considering the strong inter-hemispherical connectivity of the SAN found in this study, future work may investigate whether inter-hemispherical fibers, such as the anterior third of corpus callosum may have a role in speech articulation (De Benedictis et al., 2016). Finally, we used for the tractography analysis a deterministic approach. However, DTI is still now the most diffused algorithm for reconstruction in the clinical and surgical fields. Moreover, our diffusion MRI protocol includes a 60 encoding directions. We preferred to utilize the original tractograms of our patients, rather than merging a healthy tractography atlas to patient data, in order to maintain the patient-specific WM topography for each tumor case.

## Conclusion

Characterization of the cortical network supporting speech articulation is important to improve our understanding of this fundamental component of the language function involving planning and executing sequences of sounds to form words. Moreover, it may as well help to determine the neural correlates of speech articulation impairment following brain injuries and to minimize the chance of its resection during surgical intervention. In this study we described a multi-modal imaging approach that uses pre-surgical and intra-operative data to map at the whole-brain level, for the first time, the iFC of the SAN. This functional network includes mostly ventroposterior frontal and parieto-temporal cortical territories in both hemispheres, some of them had not been reported in previous task based fMRI studies of speech articulation, such as the SMG and Heschl’s gyri. Furthermore, considering the two major WM tracks involved in the dorsal stream language model, AF and SLF III, we determined that SLF III gives the largest contribution of fibers providing the axonal subcortical circuits structurally connecting the cortical hubs of the articulatory loop network. Finally, we found that pre-surgical rs-fMRI data offers a possible prediction of the speech articulatory network. This, however, needs further validation on a larger dataset.

Given the potential utility of these results for pre-surgical planning and for future studies investigating disturbances of the articulatory loop with different neuroimaging techniques, we provide speech articulation spatial frequency maps and speech arrest seeding points (MNI coordinates) for download^3^.

## Ethics Statement

This study was carried out in accordance with the standard pre-operative and operative protocol of the Division of Neurosurgery of “S. Chiara” Hospital for resection of brain lesions harboring critical areas. All subjects gave written informed consent in accordance with the Declaration of Helsinki.

## Author Contributions

DZ processed the data, interpreted the results, and wrote the manuscript. FCo, MD, and GF collected the data, processed the data, and interpreted the results. UR, LA, LZ, and FCh collected the data, processed the data, interpreted the results, and revised the manuscript. PA processed the data, interpreted the results, and revised the manuscript. JJ and SS processed the data, interpreted the results, and wrote and revised the manuscript.

## Conflict of Interest Statement

The authors declare that the research was conducted in the absence of any commercial or financial relationships that could be construed as a potential conflict of interest.

## Footnotes

1 http://cibsr.stanford.edu/tools/human-brain-project/artrepair-software.html

2 http://trackvis.org

3 https://r.unitn.it/en/cimec/mri/tools#data

## References

[B1] Agarwal S. Sair H. I. Yahyavi-Firouz-Abadi N. Airan R. Pillai J. J. (2016). Neurovascular uncoupling in resting state fMRI demonstrated in patients with primary brain gliomas. *J. Magn. Reson. Imaging* 43 620–626. 10.1002/jmri.25012 26201672

[B2] Baddeley A. (1990). “The development of the concept of working memory: implications and contributions of neuropsychology”, in *Neuropsychological Impairments of Short-Term Memory*, eds Vallar G. Shallice T. (Cambridge, MA: Cambridge University Press), 54–73.

[B3] Basilakos A. Rorden C. Bonilha L. Moser D. Fridriksson J. (2015). Patterns of poststroke brain damage that predict speech production errors in apraxia of speech and aphasia dissociate. *Stroke* 46 1561–1566. 10.1161/STROKEAHA.115.009211 25908457 PMC4442076

[B4] Basilakos A. Smith K. G. Fillmore P. Fridriksson J. Fedorenko E. (2017). Functional characterization of the human speech articulation network. *Cereb. Cortex* 28 1816–1830. 10.1093/cercor/bhx100 28453613 PMC5907347

[B5] Beckmann C. F. DeLuca M. Devlin J. T. Smith S. M. (2005). Investigations into resting-state connectivity using independent component analysis. *Philos. Trans. R. Soc. Lond. B Biol. Sci.* 360 1001–1013. 10.1098/rstb.2005.1634 16087444 PMC1854918

[B6] Bello L. Acerbi F. Giussani C. Baratta P. Taccone P. Songa V. (2006). Intraoperative language localization in multilingual patients with gliomas. *Neurosurgery* 59 115–123. 10.1227/01.neu.0000243290.36910.a2 16823307

[B7] Birn R. M. (2012). The role of physiological noise in resting-state functional connectivity. *Neuroimage* 62 864–870. 10.1016/j.neuroimage.2012.01.016 22245341 PMC13374118

[B8] Boschi V. Catricala E. Consonni M. Chesi C. Moro A. Cappa S. F. (2017). Connected speech in neurodegenerative language disorders: a review. *Front. Psychol.* 8:269. 10.3389/fpsyg.2017.00269 28321196 PMC5337522

[B9] Brabenec L. Mekyska J. Galaz Z. Rektorova I. (2017). Speech disorders in Parkinson’s disease: early diagnostics and effects of medication and brain stimulation. *J. Neural Transm.* 124 303–334. 10.1007/s00702-017-1676-0 28101650

[B10] Brown E. C. Muzik O. Rothermel R. Juhasz C. Shah A. K. Fuerst D. (2014). Evaluating signal-correlated noise as a control task with language-related gamma activity on electrocorticography. *Clin. Neurophysiol.* 125 1312–1323. 10.1016/j.clinph.2013.11.026 24412331 PMC4035421

[B11] Brown E. C. Muzik O. Rothermel R. Matsuzaki N. Juhasz C. Shah A. K. (2012). Evaluating reverse speech as a control task with language-related gamma activity on electrocorticography. *Neuroimage* 60 2335–2345. 10.1016/j.neuroimage.2012.02.040 22387167 PMC3321121

[B12] Chang E. F. Wang D. D. Perry D. W. Barbaro N. M. Berger M. S. (2011). Homotopic organization of essential language sites in right and bilateral cerebral hemispheric dominance clinical article. *J. Neurosurg.* 114 893–902. 10.3171/2010.11.JNS10888 21235314

[B13] Coello A. F. Moritz-Gasser S. Martino J. Martinoni M. Matsuda R. Duffau H. (2013). Selection of intraoperative tasks for awake mapping based on relationships between tumor location and functional networks. *J. Neurosurg.* 119 1380–1394. 10.3171/2013.6.JNS122470 24053503

[B14] Cogan G. B. Thesen T. Carlson C. Doyle W. Devinsky O. Pesaran B. (2014). Sensory-motor transformations for speech occur bilaterally. *Nature* 507 94–98. 10.1038/nature12935 24429520 PMC4000028

[B15] Cordella C. Dickerson B. C. Quimby M. Yunusova Y. Green J. R. (2017). Slowed articulation rate is a sensitive diagnostic marker for identifying non-fluent primary progressive aphasia. *Aphasiology* 31 241–260. 10.1080/02687038.2016.1191054 28757671 PMC5531197

[B16] De Benedictis A. Petit L. Descoteaux M. Marras C. E. Barbareschi M. Corsini F. (2016). New insights in the homotopic and heterotopic connectivity of the frontal portion of the human corpus callosum revealed by microdissection and diffusion tractography. *Hum. Brain Mapp.* 37 4718–4735. 10.1002/hbm.23339 27500966 PMC6867471

[B17] Donahue M. J. Hoogduin H. van Zijl P. C. Jezzard P. Luijten P. R. Hendrikse J. (2011). Blood oxygenation level-dependent (BOLD) total and extravascular signal changes and DeltaR2^∗^ in human visual cortex at 1.5, 3.0 and 7.0 T. *NMR Biomed.* 24 25–34. 10.1002/nbm.1552 21259367

[B18] Duffau H. (2015). Stimulation mapping of white matter tracts to study brain functional connectivity. *Nat. Rev. Neurol.* 11 255–265. 10.1038/nrneurol.2015.51 25848923

[B19] Duffau H. Gatignol P. Denvil D. Lopes M. Capelle L. (2003). The articulatory loop: study of the subcortical connectivity by electrostimulation. *Neuroreport* 14 2005–2008. 10.1097/00001756-200310270-00026 14561939

[B20] Duffy J. R. (2016). Functional speech disorders: clinical manifestations, diagnosis, and management. *Handb. Clin. Neurol.* 139 379–388. 10.1016/B978-0-12-801772-2.00033-3 27719858

[B21] Fernández-Miranda J. C. Wang Y. Pathak S. Stefaneau L. Verstynen T. Yeh F. C. (2015). Asymmetry, connectivity, and segmentation of the arcuate fascicle in the human brain. *Brain Struct. Funct.* 220 1665–1680. 10.1007/s00429-014-0751-7 24633827

[B22] Forman S. D. Cohen J. D. Fitzgerald M. Eddy W. F. Mintun M. A. Noll D. C. (1995). Improved assessment of significant activation in functional magnetic-resonance-imaging (Fmri) - use of a cluster-size threshold. *Magnet. Reson. Med.* 33 636–647. 10.1002/mrm.1910330508 7596267

[B23] Freyschlag C. F. Duffau H. (2014). Awake brain mapping of cortex and subcortical pathways in brain tumor surgery. *J. Neurosurg. Sci.* 58 199–213. 25418274

[B24] Fridriksson J. Yourganov G. Bonilha L. Basilakos A. Den Ouden D. B. Rorden C. (2016). Revealing the dual streams of speech processing. *Proc. Natl. Acad. Sci. U.S.A.* 113 15108–15113. 10.1073/pnas.1614038114 27956600 PMC5206517

[B25] Garyfallidis E. Brett M. Amirbekian B. Rokem A. van der Walt S. Descoteaux M. (2014). Dipy, a library for the analysis of diffusion MRI data. *Front. Neuroinform.* 8:8. 10.3389/fninf.2014.00008 24600385 PMC3931231

[B26] Hau J. Sarubbo S. Perchey G. Crivello F. Zago L. Mellet E. (2016). Cortical terminations of the inferior fronto-occipital and uncinate fasciculi: anatomical stem-based virtual dissection. *Front. Neuroanat.* 10:58. 10.3389/fnana.2016.00058 27252628 PMC4877506

[B27] Hickok G. Poeppel D. (2004). Dorsal and ventral streams: a framework for understanding aspects of the functional anatomy of language. *Cognition* 92 67–99. 10.1016/j.cognition.2003.10.011 15037127

[B28] Hickok G. Poeppel D. (2007). The cortical organization of speech processing. *Nat. Rev. Neurosci.* 8 393–402. 10.1038/nrn2113 17431404

[B29] Jenkinson M. Bannister P. Brady M. Smith S. (2002). Improved optimization for the robust and accurate linear registration and motion correction of brain images. *Neuroimage* 17 825–841. 10.1006/nimg.2002.1132 12377157

[B30] Joel S. E. Caffo B. S. van Zijl P. C. and Pekar J. J. (2011). On the relationship between seed-based and ICA-based measures of functional connectivity. *Magnet. Reson. Med.* 66 644–657. 10.1002/mrm.22818 21394769 PMC3130118

[B31] Kononen M. Tamsi N. Saisanen L. Kemppainen S. Maatta S. Julkunen P. (2015). Non-invasive mapping of bilateral motor speech areas using navigated transcranial magnetic stimulation and functional magnetic resonance imaging. *J. Neurosci. Methods* 248 32–40. 10.1016/j.jneumeth.2015.03.030 25845482

[B32] Levelt W. (1993). *Speaking: From Intention to Articulation.* Cambridge, MA: MIT Press.

[B33] Liu H. S. Stufflebeam S. M. Sepulcre J. Hedden T. Buckner R. L. (2009). Evidence from intrinsic activity that asymmetry of the human brain is controlled by multiple factors. *Proc. Natl. Acad. Sci. U.S.A.* 106 20499–20503. 10.1073/pnas.0908073106 19918055 PMC2777963

[B34] Mandonnet E. Sarubbo S. Duffau H. (2017). Proposal of an optimized strategy for intraoperative testing of speech and language during awake mapping. *Neurosurg. Rev.* 40 29–35. 10.1007/s10143-016-0723-x 27194132

[B35] Mandonnet E. and Duffau H. (2017). “Mapping the brain for primary brain tumor surgery”, in *Malignant Brain Tumors*, eds Moliterno Gunel J. Piepmeier J. Baehring J. (Cham: Springer), 63–79.

[B36] Marchitelli R. Minati L. Marizzoni M. Bosch B. Bartres-Faz D. Muller B. W. (2016). Test-retest reliability of the default mode network in a multi-centric fMRI study of healthy elderly: effects of data-driven physiological noise correction techniques. *Hum. Brain Mapp.* 37 2114–2132. 10.1002/hbm.23157 26990928 PMC6867386

[B37] Mayka M. A. Corcos D. M. Leurgans S. E. Vaillancourt D. E. (2006). Three-dimensional locations and boundaries of motor and premotor cortices as defined by functional brain imaging: a meta-analysis. *Neuroimage* 31 1453–1474. 10.1016/j.neuroimage.2006.02.004 16571375 PMC2034289

[B38] Minati L. Chan D. Mastropasqua C. Serra L. Spano B. Marra C. (2014). Widespread alterations in functional brain network architecture in amnestic mild cognitive impairment. *J. Alzheimers Dis.* 40 213–220. 10.3233/JAD-131766 24366921

[B39] Murphy K. Birn R. M. Bandettini P. A. (2013). Resting-state fMRI confounds, and cleanup. *Neuroimage* 80 349–359. 10.1016/j.neuroimage.2013.04.001 23571418 PMC3720818

[B40] Nishida M. Korzeniewska A. Crone N. E. Toyoda G. Nakai Y. Ofen N. (2017). Brain network dynamics in the human articulatory loop. *Clin. Neurophysiol.* 128 1473–1487. 10.1016/j.clinph.2017.05.002 28622530 PMC5512585

[B41] Pallud J. Zanello M. Kuchcinski G. Roux A. Muto J. Mellerio C. (2018). Individual variability of the human cerebral cortex identified using intraoperative mapping. *World Neurosurg.* 109 313–317. 10.1016/j.wneu.2017.09.170 28989049

[B42] Papagno C. Comi A. Riva M. Bizzi A. Vernice M. Casarotti A. (2017). Mapping the brain network of the phonological loop. *Hum. Brain Mapp.* 38 3011–3024. 10.1002/hbm.23569 28321956 PMC6866778

[B43] Paus T. (2005). Inferring causality in brain images: a perturbation approach. *Philos. Trans. R. Soc. Lond. B Biol. Sci.* 360 1109–1114. 10.1098/rstb.2005.1652 16087451 PMC1854935

[B44] Petrella J. R. Shah L. M. Harris K. M. Friedman A. H. George T. M. Sampson J. H. (2006). Preoperative functional MR imaging localization of language and motor areas: effect on therapeutic decision making in patients with potentially resectable brain tumors. *Radiology* 240 793–802. 10.1148/radiol.2403051153 16857981

[B45] Power J. D. Schlaggar B. L. Petersen S. E. (2015). Recent progress and outstanding issues in motion correction in resting state fMRI. *Neuroimage* 105 536–551. 10.1016/j.neuroimage.2014.10.044 25462692 PMC4262543

[B46] Price C. J. (2010). The anatomy of language: a review of 100 fMRI studies published in 2009. *Ann. N. Y. Acad. Sci.* 1191 62–88. 10.1111/j.1749-6632.2010.05444.x 20392276

[B47] Riva M. Fava E. Gallucci M. Comi A. Casarotti A. Alfiero T. (2016). Monopolar high-frequency language mapping: can it help in the surgical management of gliomas? A comparative clinical study. *J. Neurosurg.* 124 1479–1489. 10.3171/2015.4.JNS14333 26406788

[B48] Rosazza C. Minati L. Ghielmetti F. Mandelli M. L. Bruzzone M. G. (2012). Functional connectivity during resting-state functional mr imaging: study of the correspondence between independent component analysis and region-of-interest-based methods. *Am. J. Neuroradiol.* 33 180–187. 10.3174/ajnr.A2733 21998099 PMC7966157

[B49] Sanai N. Mirzadeh Z. and Berger M. S. (2008). Functional outcome after language mapping for glioma resection. *N. Engl. J. Med.* 358 18–27. 10.1056/NEJMoa067819 18172171

[B50] Sarubbo S. De Benedictis A. Merler S. Mandonnet E. Balbi S. Granieri E. (2015). Towards a functional atlas of human white matter. *Hum. Brain Mapp.* 36 3117–3136. 10.1002/hbm.22832 25959791 PMC6869563

[B51] Sarubbo S. De Benedictis A. Merler S. Mandonnet E. Barbareschi M. Dallabona M. (2016). Structural and functional integration between dorsal and ventral language streams as revealed by blunt dissection and direct electrical stimulation. *Hum. Brain Mapp.* 37 3858–3872. 10.1002/hbm.23281 27258125 PMC6867442

[B52] Saur D. Kreher B. W. Schnell S. Kummerer D. Kellmeyer P. Vry M. S. (2008). Ventral and dorsal pathways for language. *Proc. Natl. Acad. Sci. U.S.A.* 105 18035–18040. 10.1073/pnas.0805234105 19004769 PMC2584675

[B53] Seghier M. L. Price C. J. (2016). Visualising inter-subject variability in fMRI using threshold-weighted overlap maps. *Sci. Rep.* 6:20170. 10.1038/srep20170 26846561 PMC4742862

[B54] Sporns O. (2013). The human connectome: origins and challenges. *Neuroimage* 80 53–61. 10.1016/j.neuroimage.2013.03.023 23528922

[B55] Tate M. C. Herbet G. Moritz-Gasser S. Tate J. E. and Duffau H. (2014). Probabilistic map of critical functional regions of the human cerebral cortex: Broca’s area revisited. *Brain* 137 2773–2782. 10.1093/brain/awu168 24970097

[B56] Thiebaut de Schotten M. Ffytche D. H. Bizzi A. Dell’Acqua F. Allin M. Walshe M. (2011). Atlasing location, asymmetry and inter-subject variability of white matter tracts in the human brain with MR diffusion tractography. *Neuroimage* 54 49–59. 10.1016/j.neuroimage.2010.07.055 20682348

[B57] Tzourio-Mazoyer N. Landeau B. Papathanassiou D. Crivello F. Etard O. Delcroix N. (2002). Automated anatomical labeling of activations in SPM using a macroscopic anatomical parcellation of the MNI MRI single-subject brain. *Neuroimage* 15 273–289. 10.1006/nimg.2001.0978 11771995

[B58] Van Dijk K. R. Hedden T. Venkataraman A. Evans K. C. Lazar S. W. Buckner R. L. (2009). Intrinsic functional connectivity as a tool for human connectomics: theory, properties, and optimization. *J. Neurophysiol.* 103 297–321. 10.1152/jn.00783.2009 19889849 PMC2807224

[B59] van Geemen K. Herbet G. Moritz-Gasser S. Duffau H. (2014). Limited plastic potential of the left ventral premotor cortex in speech articulation: evidence from intraoperative awake mapping in glioma patients. *Hum. Brain Mapp.* 35 1587–1596. 10.1002/hbm.22275 23616288 PMC6869841

[B60] Zaca D. Agarwal S. Gujar S. K. Sair H. I. Pillai J. J. (2014). Special considerations/technical limitations of blood-oxygen-level-dependent functional magnetic resonance imaging. *Neuroimaging Clin. N. Am.* 24 705–715. 10.1016/j.nic.2014.07.006 25441509

[B61] Zaca D. Nickerson J. P. Deib G. Pillai J. J. (2012). Effectiveness of four different clinical fMRI paradigms for preoperative regional determination of language lateralization in patients with brain tumors. *Neuroradiology* 54 1015–1025. 10.1007/s00234-012-1056-2. 22744798

