# Supplementary material for: Whole-Brain Network Connectivity Underlying the Human Speech Articulation as Emerged Integrating Direct Electric Stimulation, Resting State fMRI and Tractography
Source: Front Hum Neurosci. 2018 Oct 11;12:405. doi: 10.3389/fnhum.2018.00405 (PMC6193478; doi:10.3389/fnhum.2018.00405)
Supplement: Supplementary file 1 [file Data_Sheet_1.docx]

Supplementary Material

**Whole-brain network connectivity underlying the human speech articulation as emerged integrating direct electric stimulation, resting state fMRI and tractograhy.**

Domenico Zacà^1^, Francesco Corsini^2,3^, Umberto Rozzanigo^4^, Monica Dallabona^2^, Paolo Avesani^5^, Luciano Annicchiarico^2,6^, Luca Zigiotto^2^, Giovanna Faraca^2^, Franco Chioffi^2,3^, Jorge Jovicich^1°^ and Silvio Sarubbo^2,3°*^

^1^ Center for Mind/Brain Sciences (CiMeC), University of Trento, TN, Italy.

^2^ Division of Neurosurgery, “S. Chiara” Hospital, Trento APSS, TN Italy.

^3^ Structural and Functional Connectivity Lab (SFC-Lab) Project, Division of Neurosurgery, “S. Chiara” Hospital, Trento APSS, TN, Italy.

^4^ Department of Radiology, Neuroradiology Unit, “S. Chiara” Hospital, Trento APSS, TN, Italy.

^5^ NiLab, Bruno Kessler Foundation - FBK, Trento, TN, Italy.

^6^ Department of Neurosciences, Biomedicine and Movement Sciences, Section of Neurosurgery, University of Verona, VR, Italy.

°These authors contributed equally to this work

*Correspondence to:

Silvio Sarubbo, MD PhD

[silviosarubbo@gmail.com](mailto:silviosarubbo@gmail.com)

# Supplementary Figures

We added as supplementary material three figures resuming, respectively: the detailed steps for rs-fMRI data processing, the maps representing the overlap between each speech arrest network that were used to determine a group speech arrest network and the matching between the independent component analysis and the seed-based analysis based on the direct electrical stimulation sites evoking speech arrest in each patient.

## Supplementary Figure 1


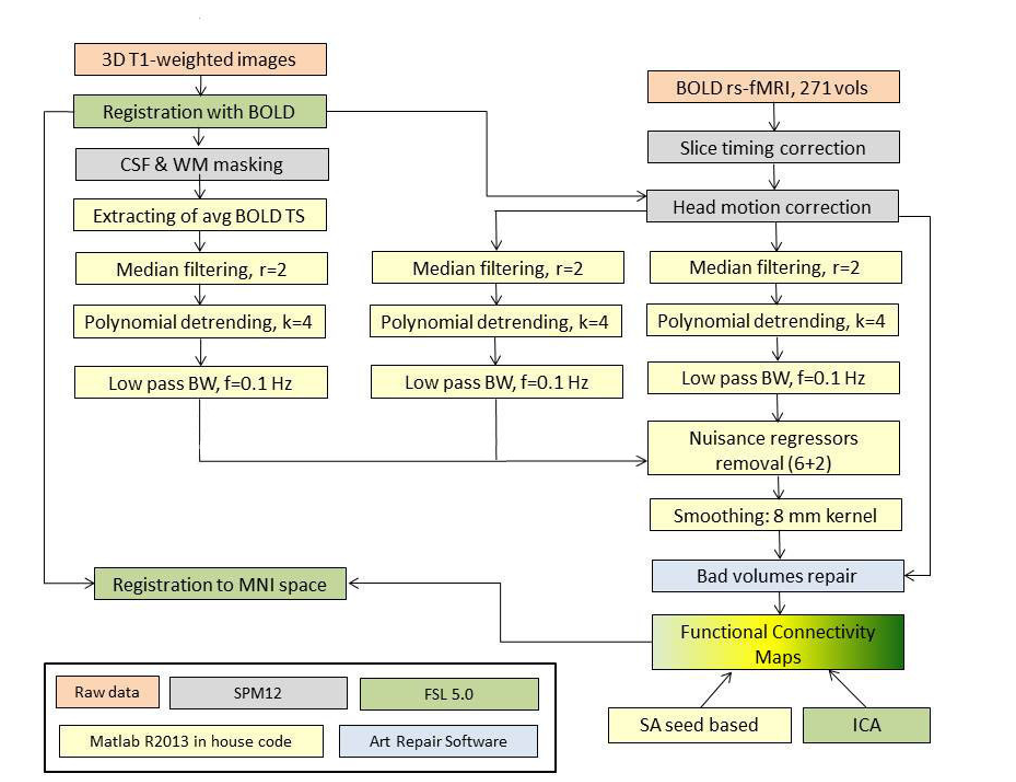


**Supplementary Figure 1.** Schematic representation of the pipeline for rs-fMRI data preprocessing.

## Supplementary Figure 2


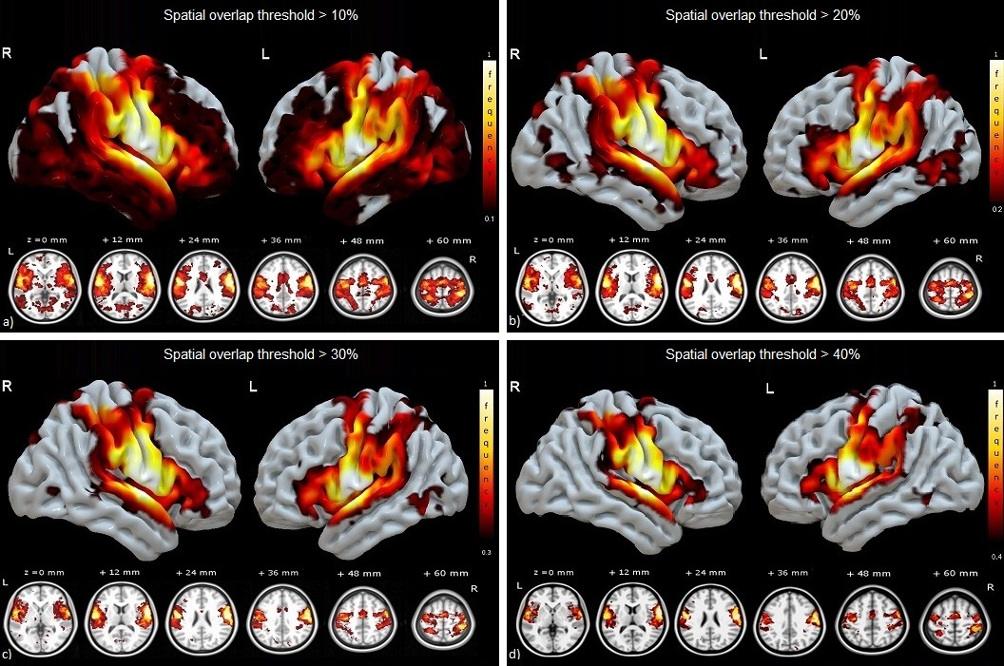


**Supplementary Figure 2**: Spatial overlap, represented as % of subjects with common SAN voxels, of the single subjects SAN in common MNI space thresholded at 4 different levels from which the group MNI SAN network (threshold 40%, Supplementary Figure 2d) shown in Figure 3 in the manuscript was determined.

## Supplementary Figure 3


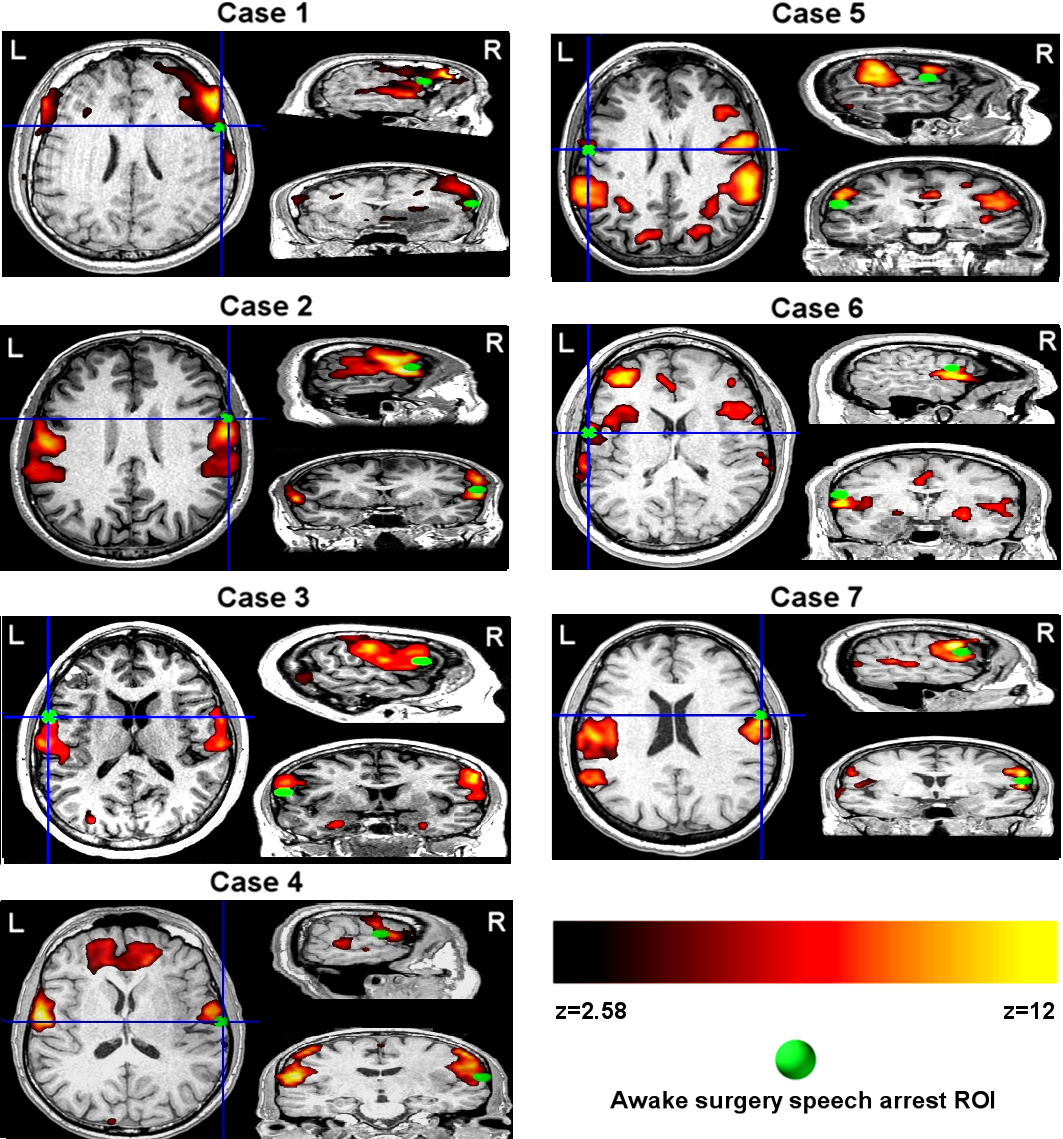


**Supplementary Figure 3.** In this boxes we report for each subject the pre-surgical resting state network (z-score map), obtained with Independent Component Analysis (ICA), with the highest spatial overlap with the speech articulation network (SAN) template obtained from the group speech articulation frequency functional connectivity map. For each patient we found overlap between the intra-operatively determined speech arrest site and the speech articulation network obtained with ICA. Data are shown in each subject’s space. L/R: left/right brain hemispheres.
